# Role of IL-25 on Eosinophils in the Initiation of Th2 Responses in Allergic Asthma

**DOI:** 10.3389/fimmu.2022.842500

**Published:** 2022-05-09

**Authors:** Bo Peng, Lin Sun, Meng Zhang, Huacheng Yan, Guochao Shi, Zhenwei Xia, Ranran Dai, Wei Tang

**Affiliations:** ^1^Department of Pulmonary and Critical Care Medicine, Ruijin Hospital, Shanghai Jiao Tong University School of Medicine, Shanghai, China; ^2^Institute of Respiratory Diseases, Shanghai Jiao Tong University School of Medicine, Shanghai, China; ^3^Shanghai Key Laboratory of Emergency Prevention, Diagnosis and Treatment of Respiratory Infectious Diseases, Shanghai, China; ^4^Department of Pediatrics, Ruijin Hospital, Shanghai Jiao Tong University School of Medicine, Shanghai, China

**Keywords:** IL-25, eosinophil, APC, allergic asthma, Th2 response

## Abstract

**Background:**

Eosinophils act as a secondary antigen-presenting cell (APC) to stimulate Th cell responses against antigens. IL-25 plays a significant role in eosinophil activation in allergic asthma. The role of IL-25 on the classic APC functions of dendritic cells has been elucidated. However, whether IL-25 facilitates eosinophils for antigen presentation is unknown.

**Objective:**

To elucidate the role of IL-25 on eosinophils antigen presenting function during allergic asthma and its related mechanism.

**Methods:**

Eosinophils from allergic asthma subjects were cultured with IL-25 and HDM to identify the co-stimulator molecules expression. Co-cultures of patient eosinophils and autologous naïve CD4^+^ T cells in the same culture system were to explore whether eosinophils had the capacity to promote Th cell differentiation in response to IL-25 engagement. In asthma mouse model, IL-25^-/-^ mice were exposed to HDM to investigate the effect of IL-25 on eosinophils during the sensitization phase. The impact of IL-25 on the capacity for eosinophils taking up antigens was evaluated. Mouse bone marrow derived eosinophils (BmEOS) were co-cultured with naïve CD4^+^T cells sorted from spleens under HDM and IL-25 stimulation to identify T cell differentiation.

**Results:**

IL-25 upregulated HLA-DR, PD-L1, and OX-40L expression on eosinophils from allergic asthma patients. IL-25 and HDM co-sensitized eosinophils promoted Th2 differentiation. In mouse model, IL-25^-/-^ mice experienced restrained allergic pulmonary inflammation and reduced eosinophils recruitment and antigen uptake capacity during the early sensitization phase. *In vitro*, IL-25 promoted antigen uptake by eosinophils. In BmEOS and naïve CD4^+^T cells co-culture, IL-25 accreted the proportion of CD4^+^Th2 cells, which was absent in CD4^+^T cells culture alone.

**Conclusion:**

Our data identify a novel role of IL-25 in enhancing eosinophils antigen uptake and co-stimulator molecules expression to induce Th2 priming in the context of allergic inflammation.

## Introduction

Allergic asthma is a major and increasingly prevalent chronic inflammatory disease of the airways which is characterized by airway infiltration of eosinophils, Th2 cells, basophils, and mast cells, together leading to mucus hypersecretion, airway wall remodeling, and airway hyperresponsiveness (1, 2). The most predominant and clearly described endotype of asthma is eosinophilic asthma, which accounts for over 50% of adults with asthma (3).

Eosinophils play pivotal roles in allergic airway inflammation, not only working in the effector phase to participate in the inflammation, but also functioning in the early phase to initiate Th2 cell responses, acting as a non-professional antigen-presenting cell (APC) (4, 5). Accumulating evidence revealed that eosinophils could express MHCII, migrate to lung and lymph nodes, process the exogenous antigen, and induce proliferative Th2 polarization (6–8). What drives the process was not elucidated yet.

IL-25, also known as IL-17E, has been shown as a key mediator in allergic asthma, strongly expressed during allergic airway inflammation (9). We previously showed that IL-17RA/RB expression on eosinophils of allergic asthmatics was significantly higher than that of atopic non-asthmatic controls and increased significantly 24 h after receiving allergen inhalation challenge (10, 11). Although the diverse roles of IL-25 on eosinophils including enhancing survival or reducing apoptosis have been described (12, 13), the effect of IL-25 on eosinophils antigen presentation is unknown. With high expression of IL-25 receptors on eosinophils in asthma and the capacity of IL-25 to facilitate classical antigen presentation cell-dendritic cells (DCs) to initiate Th2 responses (14–16), we therefore asked whether IL-25 possesses the activity to augment eosinophils to initiate Th2 responses.

Here, we investigated that IL-25 is critical in the initiation of Th2 responses by regulating co-stimulatory molecules expression and antigen processing of eosinophils. We identify that IL-25 could contribute to eosinophil-mediated Th2 polarization both in *ex vivo* murine and human studies. In the absence of IL-25, eosinophils antigen uptake capacity was hampered, resulting in ameliorated Th2 responses and alleviated airway inflammation.

## Methods

### Subjects

There were 14 subjects with mild-to-moderate allergic asthma enrolled in this study. Inclusion criteria included a physician diagnosis of allergic asthma and serum allergen-specific IgE>0.35 kUA/L. Exclusion criteria included upper respiratory infection within 4 weeks, prednisone use within 3 months, pregnancy, and other lung diseases or autoimmune diseases. All patients were taking low- or medium-dose ICS-LABA as their daily treatment and were well controlled. Detailed patient information is shown in **Table 1**. The study was approved by the Ethic Committee of Ruijin Hospital (No. 2019-YK061). All participants signed an informed consent form prior to the study.

**Table 1:** Patient demographics for *ex vivo* study.

Patient	Sex	Age	Duration of asthma(years)	Comorbidity	Total IgE(KU/L)	Der p-specific IgE(kUA/L)	Der f-specific IgE(kUA/L)	EOS%	FEV1,% predicted	FENO(ppb)
1	F	32	2.5	AR	453	52.6	67.9	2.43	121.2	29
2	F	30	1	AR, NP, D	141	9.53	8.94	5.91	127	101
3	M	19	12	AR	236	41.1	31.2	1.88	75.5	34
4	M	30	3	AR	246	33.8	30.2	2.57	93.4	23
5	M	20	1	AR	558	71.7	70.5	10.69	104.5	18
6	M	46	40	AR	130	7.25	6.84	3.06	80.9	21
7	M	24	17	AR	1234	65.3	69.3	7.73	68.9	60
8	F	20	5	AR	609	15.2	15	7.94	85	92
9	M	35	30	AR, U	511	>100	>100	3.1	82.1	108
10	M	34	1	AR	87	10.2	17.4	2.39	115.3	37
11	F	24	16	AR	284	29.6	28.9	1.85	78.8	59
12	M	39	4	AR	164	12.2	12.2	5.02	104.7	15
13	M	36	1.5	AR	105	15.4	14.8	5.38	89.2	49
14	M	26	22	AR, AC	359	35	43.2	5.6	92	75

Der p, Dermatophagoides pteronyssinus; Der f, Dermatophagoides farinase; F, female; M, male; AR, allergic rhinitis; NP, nasal polyps; D, dermatitis; U, urticaria; AC, allergic conjunctivitis; EOS%,The Percentage of Peripheral Eosinophils; FEV1, Forced Expiratory Volume in One Second; FENO,Fractional Exhaled Nitric Oxide.

### *In Vitro* Eosinophils Stimulation Assays and Co-Culture of Eosinophils and Autologous Naïve CD4^+^ T Cells

Eosinophils and naïve CD4^+^ T cells from allergic asthmatics were separated from peripheral blood by magnetic cell separation system (MACS) using Eosinophil Isolation Kit (#130-092-010, Miltenyi) and Naïve CD4^+^ T Cell Isolation Kit II (#130-094-131, Miltenyi) as previously described (17). Briefly, peripheral blood was layered on Ficoll (GE Healthcare) and centrifuged at 800 g for 20 min. Eosinophils were negatively isolated by depletion of non-eosinophils (magnetically labeled with a cocktail of antibodies and anti-biotin monoclonal antibodies conjugated to MicroBeads) from the granule cell layer suspension. The magnetically labeled non-eosinophils are depleted by retaining them on an MACS column in the magnetic field of a separator, while the unlabeled eosinophils pass through the column. Naïve CD4^+^ T cells were negatively sorted from mononuclear cell layer suspension. Purity of isolated cells was checked by flow cytometry and was always >95%. Purified eosinophils were resuspended at 1.0x10^6^/mL in RPMI 1640 (Hyclone) supplemented with 10% FBS, 1% penicillin/streptomycin (Servicebio), and 10 ng/ml IL-5 (Peprotech). Eosinophils were stimulated with 100 μg/ml house dust mite extracts (HDM, Greer Lab) and/or 1 ng/ml IL-25 (Peprotech) at 37°C in a humidified 5% CO_2_ incubator for 18 h, and then cells were harvested for co-culture or direct flowcytometry analysis.

After 18 h stimulation with HDM and/or IL-25, eosinophils (1.0x10^6^/ml) were resuspended with autologous naïve CD4^+^ T cells (10^6^/ml) in 1:1(v:v) RPMI 1640 (Hyclone) with ImunoCult-XF T Cell Expansion Medium (Stemcell) supplemented with 10 ng/ml IL-2 (Peprotech), 10 ng/ml IL-5 (Peprotech) and 25 μl/ml ImmunoCult Human CD3/CD28 T Cell Activator (stem cell). Cells were cultured at 37°C in a humidified 5% CO_2_ incubator for 48 h. Individual naïve CD4^+^ T cells (1.0x10^6^/ml) groups served as control, cultured in the same condition medium as above. Four hours before the end of culture, 1:1000 Monensin Solution (Biolegend) was added for intracellular cytokines analysis.

The flow cytometry analyses were performed with a Cytoflex LX instrument (Beckman). Dead cells were excluded by Zombie UV (Biolegend). For eosinophil surface marker analysis, cells were incubated with Fc Receptor Blocking Solution (Biolegend) to eliminate Fc receptor-mediated antibody binding, and then followed with staining of APC/Cy7 anti-human CD16 antibody (Clone:3G8, BD sciences), APC anti-human Siglec-8 antibody (Clone:7C9), PE anti-human CD40 antiobody (Clone : HB14), PE/Cyanine7 anti-human HLA-DR antibody (Clone:L243), Brilliant Violet 421 anti-human CD80 antibody (Clone:L307.4, BD sciences), BB515 anti-human CD86 antibody (Clone: FUN-1, BD sciences), PE/Cyanine7 anti-human PD-L1 antibody (Clone:29E.2A3), PE anti-human OX-40L antibody (Clone:11C3.1) for 20 min at 20°C in the dark, then cells were washed, fixed with Fixation Buffer (Biolgend) and resuspended in Cyto-last Buffer (Biolegend) until analysis.

To measure cytokines production of T cells after co-culture, following surface staining of FITC anti-human CD3 antibody (Clone: UCHT1), PE/Cyanine7 anti-human CD4 antibody (Clone: RPA-T4), cells were fixed by Fixation Buffer (Biolgend) for 20 min at 20°C avoided from light, and then washed twice with Intracellular Permeabilization Buffer. Cells were stained with APC/Fire 750 anti-human IFN-γ antibody (Clone:4S. B3), Brilliant Violet 421 anti-human IL-4 antibody (Clone: G077F6), PE anti-human IL-9 antibody (Clone : MH9A4), APC anti-human IL-17A antibody (Clone: BL168) in remaining permeabilization buffer for 30 min at 20°C in the dark, and then washed and resuspended in FACS buffer for analysis. All antibodies were purchased from Biolegend unless otherwise specified.

### Mice

IL-25-deficient mice(Il25^-/-^)were obtained from Tsinghua University, which have been described in previous studies (18, 19). The genotyping primers were as follows: forward, 5′-CTGCTCCAGTCAGCCTCTCT-3′; reverse1, 5′AGCAGCTGGGCAAGTGAC-3′; reverse2,5′ AGGTGGAGAAAGTGCCTGT-3′. All mice were bred and maintained under specific pathogen-free conditions in Shanghai Biomodel Organism Science and Technology Development Co., Ltd. Sex-matched littermate wild-type (WT) and Il25^-/-^ mice 6 to 8 weeks of age were mainly used for experiments. All animal studies were approved by the Ruijin Hospital Animal Ethics Committee.

### Mouse Model and *In Vivo* Stimulation

The HDM-induced mouse asthma model was established as described previously (20). Briefly, Il25^-/-^ and littermate WT mice were sensitized intranasally with 100 μg HDM in 40 μL PBS on Day 0 and challenged intranasally with 10 μg HDM on Days 7 to 11, harvested on Day 14. Histopathologic analysis was done to identify airway inflammation. Bronchoalveolar lavage fluid (BALF) was centrifuged and supernatants were frozen at -80°C for subsequent cytokine analysis. Cytokines IL-4, IL-5, IFN-γ, and IL-13 levels in BALF and serum IgE levels were measured by ELISA according to the manufacturer’s instructions (BioLegend).

In some experiments for investigating the role of eosinophils in the sensitization period, C57bL/6J mice were sensitized with 100 μg HDM before the first challenge and lungs were harvested for flow cytometry analysis at indicated times. To study the effect of IL-25, mice were treated intranasally with 1 μg IL-25(R&D) in 40 μL PBS and lungs were digested to obtain the single cell suspension for flow cytometry analysis 24 h later. To analyze antigen processing by eosinophil, 100 μg HDM and 50 μg DQ-OVA (Thermofisher, US) in 40 μL PBS were intranasally administrated to Il25^-/-^ and littermate WT mice. Lungs were collected and analyzed by flow cytometry 3 days later.

### Generation of Mouse Bone Marrow-Derived Eosinophil (BmEOS) and DQ-OVA Uptake by BmEOS

The *ex vivo* culture of BmEOS was adapted from the previously published protocols with minor modifications (21, 22). Briefly, bone marrow (BM) was flushed out from the femur and tibiofibula of C57bl/6J mice. After erythrocyte lysis, BM cells were seeded at 1×10^6/ml in IMDM media supplemented with 20% fetal bovine serum (Gibco), 1% penicillin/streptomycin, 2 mM L-glutamine (Gibco), 1 mM sodium pyruvate (Gibco), and 50 mM 2-mercaptoethanol (Sigma-Aldrich) in the presence of 100 ng/mL FLT-3L and 100 ng/mL recombinant murine stem cell factor (SCF, Peprotech), cultured from Day 0 to Day 4. On Day 4 and 8, the media containing SCF and FLT3-L was replaced with media containing 10 ng/mL recombinant mouse interleukin-5 (rmIL-5, R&D Systems) only. Every other day, from this point forward, media was replaced with fresh media containing rmIL-5. After Day 10, cells were seeded and used for subsequent experiments.

BmEOS were seeded in 48 wells plates and stimulated for 2 days with or without HDM and IL-25 in complete medium. To assess DQ-OVA uptake by BmEOS, BmEOS were treated with DQ-OVA for 12 h before being collected, washed, and further analyzed using flow cytometry. Cells cultured in the absence of DQ-OVA were set as a negative control.

### *In Vitro* Polarization of CD4^+^ T Cells With BmEOS

Spleens were removed from C57bl/6J 6~8-week-old mice, mechanically disrupted, treated with erythrocyte lysis buffer, and run through 70-μm cell strainer. Suspensions were resuspended in MACS buffer, labelled with an antibody cocktail, and naïve CD4^+^ T cells were negatively separated using the mouse Naïve CD4^+^ T cell Isolation kit (#130-104-453, Miltenyi), in accordance with manufacturer’s instructions. The *ex vivo* co-culture of BmEOS and CD4^+^T was adapted from the previously published literature with modifications (7). High purity isolated naïve CD4^+^ T cells (1*10^6/ml) were co-cultured with the BmEOS (5*10^5/ml) in supplemented IMDM medium with or without 100 ug/ml HDM in the presence or absence of 50 ng/ml IL-25, with 10 ng/ml IL-2 and 10 ng/ml GM-CSF (R&D) treatment to sustain T cells survival. As controls, CD4^+^ T cells alone were also cultured. Cells were incubated for 96 h at 37°C in a 5% CO_2_ humidified atmosphere. During the last 5 h, cells were re-stimulated with cell stimulation cocktail (combined with phorbol 12-myristate 13-acetate (PMA), ionomycin, brefeldin A, and monensin) (eBioscience #00-4975-03) and then were harvested and stained with fluorescent antibodies for flow cytometric analysis.

### Preparation of Single-Cell Suspension for Flow Cytometry

BALF was collected from lungs *via* two intratracheal infusions with 0.8 ml PBS using a 20G flushing needle. To obtain single-cell suspensions from lung tissues, lungs were intubated and digested for 1 h at 37°C with intermittent pipetting every 15 min in Hank’s Balanced Salt Solution(HBSS, Invitrogen) media containing 1 mg/mL type 1A collagenase (Sigma) and 30 μg/mL DNase I (Sigma). The digested lung fragments were passed through 70 μm filters, treated with erythrocyte lysis buffer, washed, and resuspended in PBS with 1% FBS.

### Flow Cytometry

For surface staining, single-cell suspensions were incubated with fluorochrome-conjugated antibodies cocktails for 30 min at 4°C. For intracellular cytokine staining, cells were fixed and permeabilized using Fixation/Permeabilization Buffer Set (eBioscience) according to the manufacturer’s protocol. Fluorochrome-conjugated anti-mouse mAbs used in these experiments included the following: CD45-BV510, Siglec-F-AF647, CD11c-FITC, CD11b-PECY7, CD4-PECY7, IL-4-BV421, IL-17A-AF647, IFN-γ-FITC, IL-9-PE, CD44-FITC (Miltenyi), CD62L-APC, and CD3-PERCPCY5.5. A fixable viability dye eF780 was used to distinguish viable cells from nonviable cells. All antibodies were purchased from BD Biosciences unless otherwise specified. Flow cytometry data were collected using BD Fortessa or Beckman Coulter CytoFlex S and analyzed by FlowJo X software.

### Data Analysis

Flow cytometry data were analyzed by CytExpert (Beckman, US) or FlowJo X software. Statistical significance was performed by analysis of one-way ANOVA with *post-hoc* Holm-Sidak’s test or a two-sided unpaired Student’s t-test. Statistical analysis was performed using Graphpad Prism 8.0 (Graphpad software, US). All data were presented as mean ± SD. P values less than 0.05 were considered statistically significant.

## Results

### IL-25 and HDM Cooperated to Upregulate HLA-DR, PD-L1, and OX-40L Expression on Eosinophils From Allergic Asthma Patients

**Figure 1:**
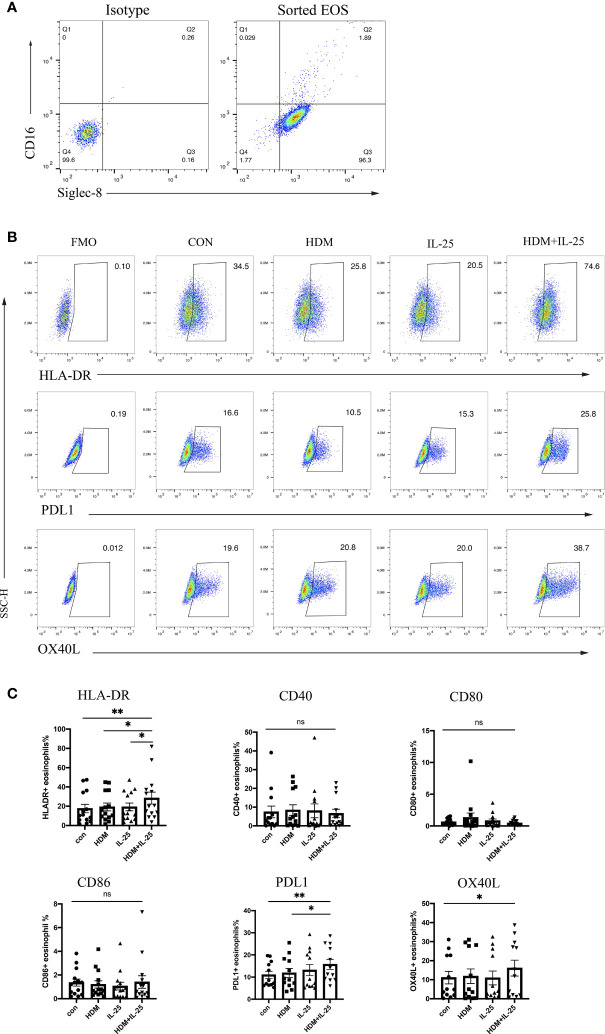
IL-25 and HDM co-promote expression of surface HLA-DR, PD-L1, and OX-40L on eosinophils. **(A)** FACS plots of the purity for eosinophils (CD16^-^Siglec^-^8^+^ cells) after sorting. **(B)** Gated on viable eosinophils (Zombie^-^CD16^-^Siglec-8^+^ cells), expression rates of CD40, HLA-DR, CD80, CD86, PD-L1, and OX-40L in the presence or absence of HDM and/or IL-25 were tested by flow cytometry. **(C)** Graphs showed expression rates of CD40, HLA-DR, CD80, CD86, PD-L1, and OX-40L on eosinophils from allergic asthma patients. n = 14, ns, no significant difference, ^**^p < 0.01, ^*^p < 0.05.

Effective antigen presentation requires expression of both MHC-II and co-stimulatory molecules on APCs, so we first examined whether IL-25 had an impact on the expression of those co-stimulatory molecules of eosinophils from allergic asthmatics *in vitro*. We sorted eosinophils from peripheral blood and confirmed the purity (**Figure 1A**). When stimulated with both HDM and IL-25 for 18 h, the expression of HLA-DR, PD-L1, and OX-40L of cultured eosinophils was found to be significantly increased compared with the control group. HLA-DR expression was also elevated in the HDM+IL-25 group compared to HDM or IL-25 alone group. The expression of PD-L1 was significantly higher when cultured with HDM+IL-25 than HDM alone (**Figures 1B, C**).

### IL-25 and HDM Co-Sensitized Eosinophils Promoted Th2 Differentiation of Autologous Naïve CD4^+^ T Cells From Allergic Asthma Patients

**Figure 2:**
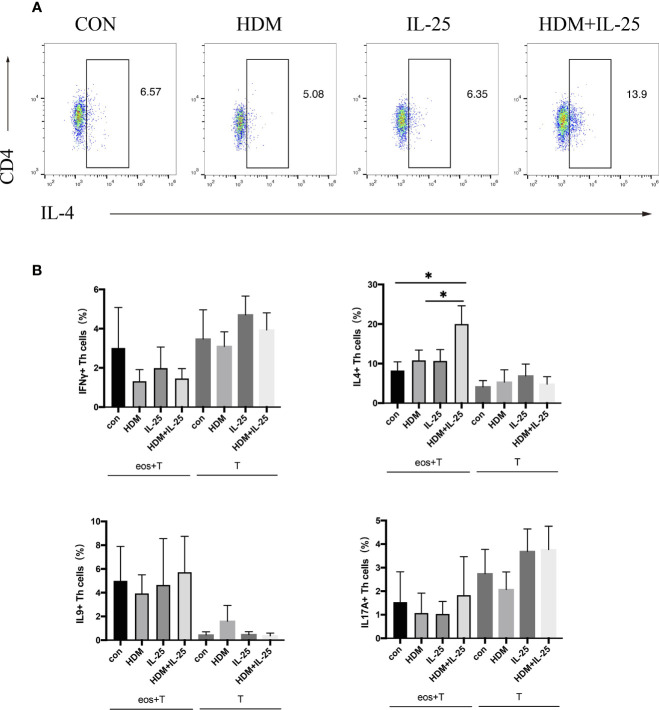
IL-25 plus HDM-sensitized eosinophils promote Th2 differentiation of autologous naïve CD4^+^ T cells from allergic asthma patients. **(A)** Gated on viable CD4^+^ T cells (Zombie^-^CD3^+^CD4 ^+^cells), expression of intracellular IFN-γ, IL-4, IL-9, and IL-17A were tested by flow cytometry. **(B)** Graphs show percentages of intracellular IFN-γ, IL-4, IL-9, and IL-17A expression on CD4^+^ T cells in co-culture system or cultured alone. n = 5, ^*^p < 0.05.

Eosinophils were pulsed with HDM and/or IL-25 for 18 h, and then went through a co-culture procedure with autologous naïve CD4^+^ T cells for up to 48 h. Intracellular cytokines expression was tested by flow cytometry. Signature effector cytokines IFN-γ, IL-4, IL-9, and IL-17A were selected to stand for Th1, Th2, Th9, and Th17 cells, respectively. When treated with both IL-25 and HDM, eosinophils further promoted differentiation of autologous naïve CD4^+^ T cells toward Th2 cells, which was unseen in IL-25 or HDM mono-stimulation group. Comparatively, in groups where eosinophils were absent, IL-25 and HDM separately were unable to promote Th cell differentiation effectively (**Figure 2**).

### IL-25 Was Required for Induction of Allergic Airway Inflammation

**Figure 3:**
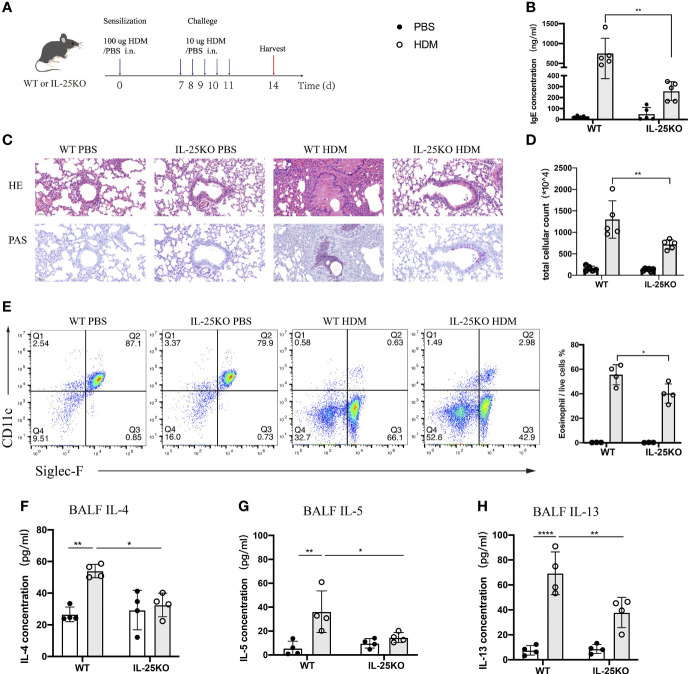
IL-25 deficiency ameliorates HDM-induced airway inflammation. **(A)** Schematic representation of house dust mite (HDM)-induced allergic airway inflammation of wild-type and global IL-25^-/-^ mice. **(B)** Serum IgE level after HDM or PBS challenge. **(C)** HE&PAS lung histology images of wild-type and global IL-25^-/-^ mice after PBS or HDM challenge. Scale bars: 50 μm. **(D)** Absolute cellularity of total cells in BALF of mice in **(A)**. **(E)** Flow cytometry analyzed the percentages of BALF eosinophils (CD45^+^SiglecF^+^CD11c^-^) infiltration into airways after HDM or PBS challenge. **(F–H)** BALF IL-4 **(F)**, IL-5 **(G)**, and IL-13 **(H)** cytokine concentration of mice as in **(A)**. n= 4~5, ANOVA **(B, D, E–H)**, *p < 0.05, **p < 0.01, ****p < 0.0001.

To determine whether IL-25 is essential for induction of allergic airway inflammation, we subjected IL-25-deficient (Il25^-/-)^ and littermate wild-type mice to allergic airway inflammation induced by HDM, the most common allergen of allergic asthma in Chinese patients (23) (**Figure 3A**). After a sensitization and challenge phase with HDM, WT mice developed a severe lung pathology, increased serum IgE, and high Th2-cell-mediated inflammatory responses. We found that Il25^-/-^ mice, compared with WT mice, had an alleviated airway pathology *via* H&E staining and periodic acid-Schiff (PAS) assay (**Figure 3C**), decreased IgE production (**Figure 3B**) and cellular infiltration in the airways (**Figure 3D**), less eosinophils (**Figure 3E**), and reduced type 2-cytokines level, including IL-4, IL-5, and IL-13 (**Figures 3F–H**). These data suggest that IL-25 is required to maintain disease severity during allergen-mediated Th2 immune responses.

### IL-25 Induced Eosinophil Recruitment in the Sensitization Phase

**Figure 4:**
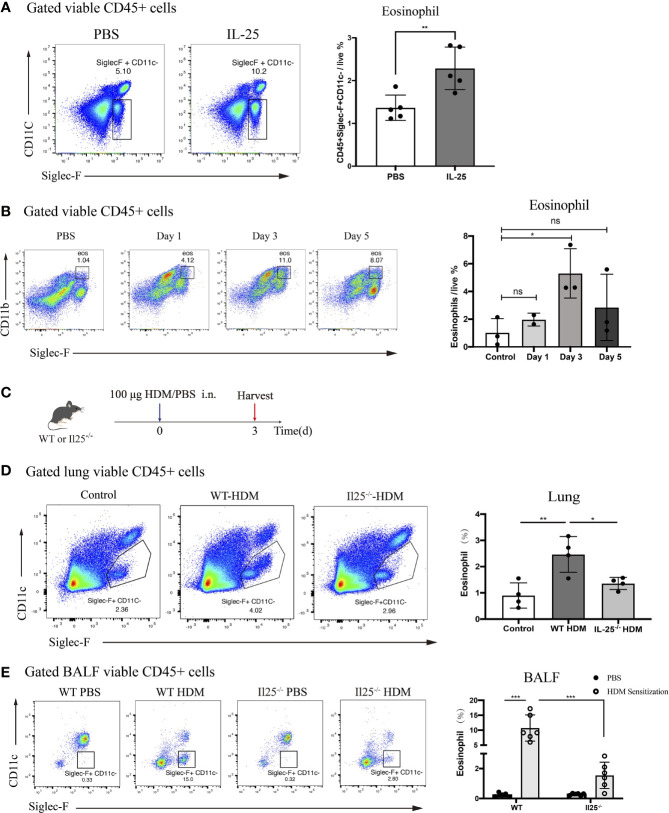
IL-25 induces eosinophils recruitment after HDM sensitization *in vivo*. **(A)** Mice were instilled intranasally with 100 ug/40 μL murine recombinant IL-25 or 40 μL PBS. After 1 day, mice were sacrificed for analyzing lung eosinophils. Flow plots (left) show the extent of eosinophils (viable CD45^+^SiglecF^+^CD11c^-^) infiltration into the lungs of mice after IL-25 exposure. The bar graph (right) shows the percentages of eosinophils infiltration after IL-25 exposure. Each dot represents a mouse. **(B)** Flow cytometry analysis of eosinophils infiltration into lungs after 100 μg/40 μL HDM or 40 μL PBS sensitization of WT mice at indicated times. **(C)** Schematic representation of mice 3 days after HDM priming. **(D, E)** Analysis of the percentages of eosinophils infiltration into lungs **(D)** and airways **(E)** of WT and Il25^-/-^ mice 3 days after HDM or PBS sensitization as in **(C)**. n=5 for **(A)**; n=2∼3 for **(B)**; n = 4 for **(D)**; n = 6 for **(E)** Unpaired Student's test for **(A, B, D)**; one-way ANOVA for **(E)**; ns, no significant difference, *p < 0.05, **p < 0.01, ***p < 0.001.

IL-25 was shown in previous research to directly activate conventional DCs *in vivo* (24). Thus, we sought to determine the effect of IL-25 *in vivo* on eosinophils. We administered recombinant murine IL-25 (1 μg/mouse) or PBS to WT mice. After 24 h, lungs were harvested and analyzed for infiltrated eosinophils. There were significantly more eosinophils in lungs of IL-25-treated mice compared with PBS-treated control mice (**Figure 4A**). Thus, IL-25 promotes eosinophils to recruit to lungs *in vivo*.

Previous studies have identified eosinophils process antigen to promote Th2 cells expansion (6–8). Since the antigen-presenting role occurs during the sensitization phase, we wondered whether IL-25 affects eosinophils during the sensitization phase. A sensitized dose of HDM was administrated intranasally into WT mice and we explored changes in lung eosinophils from day 0 to day 5.We found that eosinophils were significantly increased on the third day after HDM sensitization, suggesting it may exert an antigen-presenting role at this time (**Figure 4B**). To determine whether IL-25 contributes to the role of eosinophils in sensitization response, we analyzed eosinophils in IL-25^-/-^ mice 3 days after HDM sensitization (**Figure 4C**). IL-25^-/-^ mice exhibited reduced eosinophils compared with WT mice, both in lung and BALF (**Figures 4D, E**), which demonstrated IL-25 was required in promoting eosinophils recruitment to airway to exert its role in the sensitization phase, before the onset of the effector stage.

### IL-25 Deficiency Reduced Mouse Antigen Uptake by Pulmonary Eosinophils *In Vivo*

**Figure 5:**
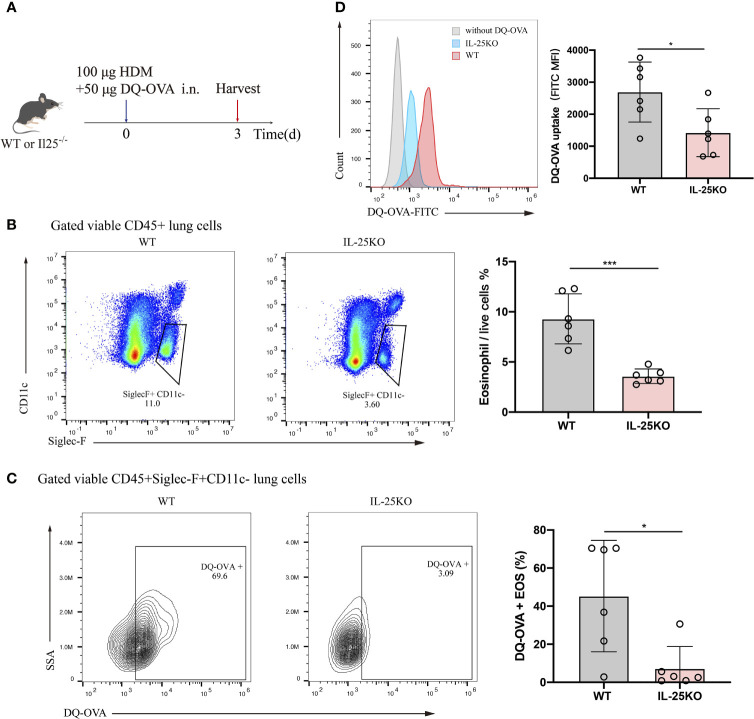
IL-25 deficiency reduced mouse antigen uptake by pulmonary eosinophil *in vivo*. **(A)** Schematic representation of IL-25KO and littermate WT mice receiving traceable DQ-OVA together with HDM injection intranasally (n = 6 per group), followed by an analysis on day 3. **(B)** The flow plots and statistical analysis of eosinophils infiltration in lungs of mice as in **(A)**. **(C)** Analysis for DQ-positive eosinophils in the lung of mice as in **(A)**. **(D)** MFI values of DQ-OVA for eosinophils in the lung of mice as in **(A)**. n = 6. Unpaired Student’s t test **(B–D)**, *p < 0.05, ***p < 0.001.

Considering the predominant role for antigen uptake in antigen-presenting cells, DQ-OVA, which becomes fluorescent upon processing by antigen-presenting cells, was co-administered intranasally with HDM into IL-25KO and littermate WT mice. Antigen processing by eosinophil was analyzed 72 h later (**Figure 5A**). IL-25^-/-^ mice displayed reduced lung eosinophils (**Figure 5B**) and DQ-labelled eosinophils (**Figure 5C**) compared to their littermate WT mice. Also, IL-25KO mice had lower mean fluorescence intensity (MFI) for eosinophils than WT mice, indicating that IL-25 deficiency affected DQ-OVA uptake in HDM-treated mice (**Figure 5D**). The data suggested that IL-25 promotes antigen uptake by eosinophils *in vivo*.

### IL-25 Promoted Antigen Uptake by Bone Marrow-Derived Eosinophils *In Vitro*

**Figure 6:**
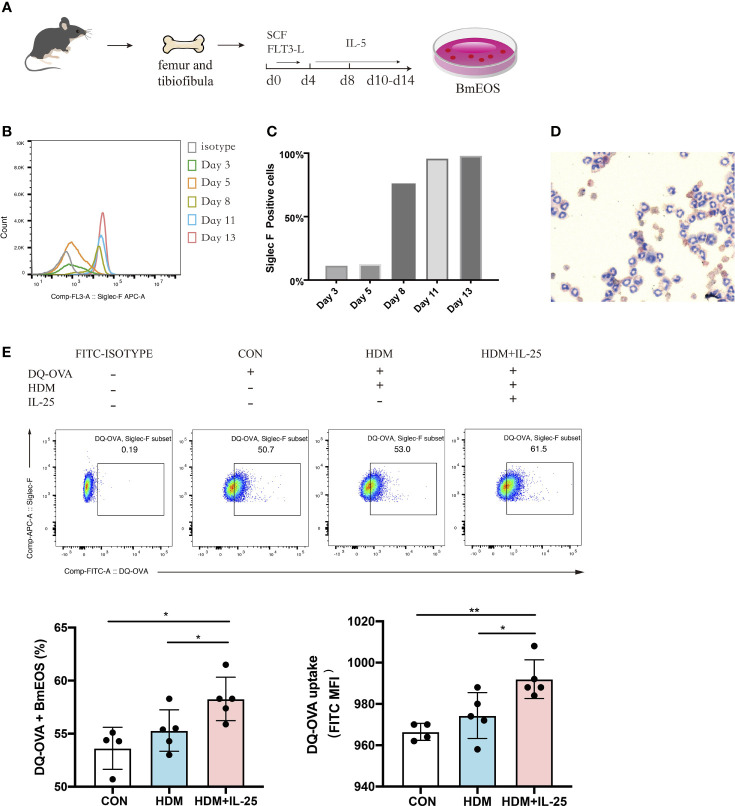
IL-25 promotes antigen uptake by BmEOS. **(A)** Flowchart of isolation and culture of BmEOS. **(B–D)** Identification of BmEOS. Histogram of Siglec-F expression at indicated times of mouse BmEOS culture **(B)**. Flow cytometry analysis of the percentages of Siglec-F positive BmEOS using isotype control and anti-Siglec-F AF647-conjugated antibody **(C)**. Light microscopic image of BmEOS at culture day 11, Diff-Quick staining **(D)**. **(E)** At day 10~13, BmEOS were treated with or without HDM in the absence or presence of IL-25 for 48 h. During the last 12 h, DQ-OVA was added. The percentages of DQ-OVA^+^ BmEOS and MFI of DQ-OVA were analyzed by flow cytometry. Representative graphs, statistical data, and MFI values are shown. Cells without DQ-OVA were set as isotype. n = 4∼5, unpaired Student’s t test **(E)**, *p < 0.05, **p < 0.01.

Next, we generated eosinophils from murine bone marrow (**Figure 6A**) and identified that BmEOS became mature and reached at high purity after Day 10 (**Figures 6B–D**). To assess whether IL-25 modulated the ability of eosinophils to uptake antigen *in vitro*, we treated BmEOS with DQ-OVA when pulsed with or without HDM. When pulsed with HDM, the uptake ability of BmEOS slightly raised but without significant statistical difference compared with the control group. Pulsed with HDM and IL-25 significantly increased uptake of DQ-OVA by BmEOS, as compared to pulsed with HDM alone (**Figure 6E**). These data suggested that IL-25 promoted the ability of eosinophils to take up the antigen *in vitro*.

### IL-25 Facilitated Th2 Cell Differentiation Induced by Eosinophils

**Figure 7:**
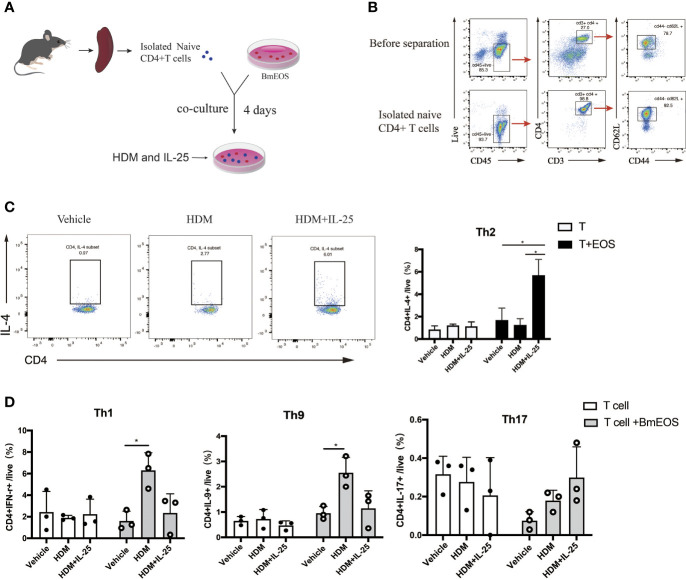
IL-25-activated eosinophils promote Th2 polarization *in vitro*. **(A)** Schematic diagram of isolated naïve CD4^+^ T cells from mice and co-culture of T-BmEOS system. **(B)** Flow cytometry identification of isolated naïve CD4^+^T cells (viable CD45^+^CD3^+^CD4^+^ CD62L^+^CD44^-^). **(C, D)** Purified naïve CD4^+^ T cells and BmEOS were co-cultured for 96 h with HDM in the presence or absence of IL-25. Naïve CD4^+^ T cells alone were also cultured. The differentiation of Th subsets was detected by flow cytometry. n=3, unpaired Student’s t test **(C, D)**, *p < 0.05.

To further investigate the effect of IL-25 on eosinophils in initiating Th cell responses, BmEOS from WT mice and naïve CD4^+^ T cells from spleens were co-cultured in the absence or presence of HDM and IL-25 (**Figures 7A, B**). The results showed that the percentage of CD4^+^ IL-4^+^ Th2 cells was significantly increased in BmEOS-T co-culture system with HDM plus IL-25 treatment, as compared with HDM treatment alone. When culturing naïve CD4^+^T cells alone, there were no detectable differences in the percentage of CD4^+^ IL-4^+^ Th2 cells among vehicle-treated, HDM-treated, and HDM plus IL-25-treated T cells, suggesting that HDM plus IL-25 treatment did not lead to CD4^+^ Th2 cells-intrinsic differentiation (**Figure 7C**). In addition, HDM-treated co-cultures developed an increase in the percentages of CD4^+^IFN-γ^+^Th1 cells and CD4^+^IL-9^+^Th9 cells than the vehicle-treatment group. However, there were no significant differences in the proportions of Th1, Th9, and Th17 cells between HDM treatment and HDM plus IL-25 treatment group, no matter in BmEOS-T co-culture or cultures of T cells alone (**Figure 7D**). These led to the conclusion that IL-25 could promote eosinophil-induced Th2 cell polarization.

## Discussion

Here we show that IL-25 has a significant role in type 2 immunity through regulating eosinophils to initiate Th2 responses. Our data demonstrated that IL-25 promotes not only eosinophils increase in lung, but also antigen uptake by eosinophils, along with eliciting the expression of surface co-stimulatory molecules on eosinophils, which together activate naïve CD4^+^ T cells polarization to Th2 cells, resulting in allergic airway inflammation ultimately.

Previous studies showed that IL-25 is critical in type 2 immune response by stimulating Th2 and ILC2 cells (25, 26). Using an *in vivo* mouse model of airway inflammation, we showed that Il25^-/-^ mice displayed mitigated inflammatory responses and Th2 responses in HDM-induced asthma model, which was consistent with the previous OVA-induced studies or anti-IL-25 administrated mice model (25, 27). Our results stressed the importance of IL-25 in allergic airway inflammation again. The contribution of IL-25 on asthma symptoms by its direct action on lung endothelial cells, DCs, and Th2 cells has been well documented (15, 25, 28, 29). During allergic airway inflammation, the receptor for IL-25 was shown to be expressed by eosinophils, suggesting a direct function of IL-25 on it (30). Diverse functions of IL-25 on eosinophils have been elucidated, including prompting cell activation, cytokine release, and survival maintenance (12), enhancing its role as effector cells in immunity responses.

It is now clear that eosinophils are more than just terminally differentiated effector cells (31) and have antigen-presenting cells-like functions, such as expression of co-stimulatory molecules (32), migration toward regional lymph nodes (33), and assistant in the priming of naïve T cells to initiate Th2 responses (7, 34, 35). The evolving recognition of the capacity of eosinophils to engage in interaction with CD4 lymphocytes and other immunologic cells suggests that eosinophils could function as antigen-presenting cells to elicit antigen-specific responses, which places eosinophils upstream of Th2 cell-mediated effector responses (36, 37).

Antigen present cells must be able to internalize foreign antigen efficiently and coordinately regulate antigen uptake, migration, and T cell stimulatory capacity (38). Antigen uptake and process is a crucial step in the initiation of antigen-specific responses. Enhanced allergen capturing and processing by airway APC populations increases T cell activation and contributes to allergic airway disease (39). In our study, IL-25 significantly promoted antigen uptake by eosinophils, supporting the idea that IL-25 can modulate antigen present capability of eosinophils. Meanwhile, we investigated that IL-25 is implicated in the priming and activation of eosinophils on their accumulation to lung. We also found that IL-25 treatment upregulated cell surface expression of HLA-DR, PD-L1, and OX40L on eosinophils in peripheral blood from allergic asthma patients. PD-L1, a co-stimulatory molecule of the CD28/B7 family, is highly expressed on dendritic cells and upregulates upon antigen uptake. PD-L1 plays a crucial role in migration and activation of antigen present cells (40). OX40L is the tumor necrosis factor receptor (TNFR) superfamily, expressed on professional and non-professional APCs. It has been shown that interaction between membrane OX40L on APCs and OX40 on naive T cells contributes to the induction T cells polarization into the Th2 subset (41, 42). It was reported in several studies that eosinophils could express CD40, CD80, and CD86 (32, 33), but we did not observe any significant increase in the expression of these surface molecules upon IL-25 stimulation, which indicates probably the role of IL-25 in promoting the antigen presentation of eosinophils is independent of these molecules.

In co-culture studies aimed at exploring the role of IL-25 on eosinophils in priming T cells, we determined that IL-25 activated-eosinophils promote naive CD4^+^ T cells differentiation to Th2 cells only but not Th1, Th17, and Th9 cells. When T cells culture alone, it has not seen IL-25 activated T cells directly, which is consistent with that reported by Yui-Hsi et al., in which they observed that IL-25 alone could induce neither Th2 polarization nor phenotypic changes of Th2 memory cells, but could induce the expansion of Th2 cells driven by DCs (43). Besides, we have observed that when HDM was added alone in the human EOS-T co-cultural system, eosinophils were not capable to induce T cells polarization. This result is consistent with the previous study that human peripheral blood eosinophils pulsed with HDM drove Th cell proliferation, but the cytokine responses were not biased toward any specific Th subset (17). However, these results differ slightly in co-culture of mouse-derived EOS and spleen T cells, in which HDM pulsed eosinophils promote Th1 and Th9 polarization. This discrepancy may be explained by cell source difference, since human experiments used cells from patients with asthma but animal experiments used cells from naive mice, with more likely complex mechanisms in patient cells. In addition, although IL-25 increased significantly Th2 cells in EOS-T co-culture systems, indicating polarization of naïve T cells, it is also likely that the increased Th2 cells results from expansion from a few existing Th2 cells in all conditions, which probably represents a different mechanism and needs further research in the future.

Our study suggested that IL-25 can facilitate naïve CD4^+^T cells differentiation to Th2 cells as we demonstrated employing mouse-derived cells and human peripheral blood eosinophils both. However, we were unable to obtain eosinophils from human airway or local lung tissues to prove it directly. Although we have uncovered multiple evidence for IL-25 enhancing eosinophils capacity in antigen presentation, the powerful role of DC as the classic antigen-presenting cell during airway inflammation is still the main pathway in initiating Th2 response. Further studies are required to define the relationship in antigen presenting function of eosinophils with or without the condition of excluding DCs *in vivo*.

In summary, we demonstrated a novel role of IL-25 on eosinophils in the initiation of type 2 immunity during allergic airway inflammation. Identifying diverse roles of eosinophils playing in the immune responses and its related triggers provides important insights and precise targets to eosinophil-associated diseases.

## Data Availability Statement

The original contributions presented in the study are included in the article/supplementary material. Further inquiries can be directed to the corresponding authors.

## Ethics Statement

The studies involving human participants were reviewed and approved by the Ethic Committee of Ruijin Hospital. The patients/participants provided their written informed consent to participate in this study. The animal study was reviewed and approved by Ruijin Hospital Animal Ethics Committee.

## Author Contributions

BP and LS conducted most of the experiments, completed the data, and contributed equally to this work. MZ offered assistance in animal model preparation and experimental operation details. HY was involved in patient-related experiments including specimen acquisition, handling, and analysis. GS made substantial contributions to the design and conduct of the experiment. ZX provided the indispensable experimental platform and constructive guidance. WT and RD conceived the project and led the study design, supervised the study, reviewed the manuscript, and provided final confirmation of the revised version to be published. All authors have read and approved the final version of the manuscript.

## Funding

Supported by the National Natural Science Foundation of China (81870021); Shanghai Municipal Key Clinical Specialty (shslczdzk02202); National Key R&D Program of China (2018YFC1311900); and Shanghai Municipal Commission of Health and Family Planning Project (201740032); Shanghai Key Laboratory of Emergency Prevention, Diagnosis and Treatment of Respiratory Infectious Diseases (20dz2261100).

## Conflict of Interest

The authors declare that the research was conducted in the absence of any commercial or financial relationships that could be construed as a potential conflict of interest.

## Publisher’s Note

All claims expressed in this article are solely those of the authors and do not necessarily represent those of their affiliated organizations, or those of the publisher, the editors and the reviewers. Any product that may be evaluated in this article, or claim that may be made by its manufacturer, is not guaranteed or endorsed by the publisher.

## Abbreviations

APC, antigen present cell; BALF, bronchoalveolar lavage fluid; BM, bone marrow; BmEOS, bone marrow derived eosinophils; DC, dendritic cell; EOS, eosinophil; HDM, house dust mite; IL, Interleukin; Il25^-/-^, IL-25-deficient; MHCII, class II major histocompatibility complex; Th2, T helper Type 2; WT, wild type.
